# Patient complaints about health care in a Swedish County: characteristics and satisfaction after handling

**DOI:** 10.1002/nop2.54

**Published:** 2016-05-10

**Authors:** Charlotta Skålén, Lena Nordgren, Eva‐Maria Annerbäck

**Affiliations:** ^1^ Patient Advisory CommitteeSörmland County CouncilNyköpingSweden; ^2^ Centre for Clinical Research SörmlandUppsala UniversityEskilstunaSweden; ^3^ Department of Public Health and Caring SciencesUppsala UniversityUppsalaSweden

**Keywords:** Patient advisory committee, patient complaints, patient safety, patient satisfaction, quality of health care, Sweden

## Abstract

**Aim:**

To describe patient complaints and to examine possible associations between healthcare providers’ statements and reports of satisfaction/dissatisfaction.

**Design:**

A retrospective and descriptive design was used to examine filed complaints.

**Methods:**

Complaints from one Patient Advisory Committee in Sweden in 2011 was examined using three different protocols/reading guides (*n = *618). Associations between contents in responses from healthcare providers and reports of satisfaction/dissatisfaction from the complainants were analysed.

**Results:**

Less than one‐third of the complainants were satisfied after handling and with healthcare providers’ statements about the complaint. The most frequent causes for dissatisfaction were that the healthcare provider ‘did not tell the truth’ or ‘gave insufficient information’. There was a statistically significant association with dissatisfaction if the statement from the healthcare provider included the category ‘disagree/defend themselves’. Four categories were associated with being satisfied and the associations were statistically significant when two or more of these were combined.

## Introduction

Patient complaints are considered a valuable source for quality improvement in health care (Reader *et al*. 2014). Moreover, patient satisfaction is an important issue for healthcare providers to fulfil their caring duties, to ensure patient safety and also for compassionate clinical nursing (Allan *et al*. 2015). In Sweden, the number of healthcare complaints is growing despite an increased patient focus in health care. In 2015 a new law was enrolled in Sweden – *The Patient Act* (2014:821) – that aims to reinforce and clarify the patients’ position and facilitate patients’ integrity, self‐determination and participation. The legislation has long been preceded by efforts in health care to achieve a higher degree of patient‐centered care. According to the act, patients are to be informed about their condition and available treatments. The patients also have the right to participate in all decisions about care. As a consequence, many Swedish healthcare organizations are currently making changes to clinical practice. Common complaints from patients concern (perceived) disrespect, disagreements over treatment, insufficient information, lack of confidence, that physicians are unavailable and lack of communication (Wofford *et al*. 2004). In Sweden, 85 per cent out of the total number of complaints of health care are addressed to patient advisory committees [PACs] (Kent 2008). The PACs have two major assignments: to help and support patients on the basis of their comments and complaints and to contribute to quality improvements in patient care. PACs do not exercise authority against healthcare personnel and cannot distribute warnings or withdraw licenses. The PACs’ assignment to help and support patients is often achieved by providing community information about patient insurance and the possibility to lodge complaint also to the National Board of Health and Welfare (or since 2013 to the Health and Social Care Inspectorate). These authorities examine health care professional's right to practice and if a licensed practitioner, for example, a physician or a nurse has failed, their license can be withdrawn. In total, the PACs in Sweden receive approximately 30,000 complaints each year. In the last four years the number has increased by 17% Despite the increase, the complaints concern less than one per thousand of all patient visits (Wessel *et al*. 2012). Subsequently, it has been argued that complaints filed at PACs are only ‘the tip of an iceberg’ (Wessel *et al*. 2012).

### Background

Research about patient complaints are conducted from different perspectives. From a nursing perspective, human suffering can be understood as the main motive for care (Eriksson 2002). Sometimes, however, care turns out to be a cause for human suffering (Eriksson *et al*. 2006). Previous studies show that patients can perceive care, caregivers or healthcare organizations as incomprehensible, strange, ambiguous and unclear leading the patients to lose trust and faith in both caregivers and organizations (Eriksson and Svedlund 2007, Nordgren *et al*. 2007, Soderberg *et al*. 2012, Wessel *et al*. 2013). Frequently, such experiences come from experiences of insufficient communication, of patients being disrespectfully encountered, objectified, depersonalized, ignored or from feelings of powerlessness (Arman *et al*. 2004, Eriksson & Svedlund 2007, Nordgren *et al*. 2007, Reader & Gillespie 2013, Skar & Soderberg 2012, Soderberg *et al*. 2012, Wessel *et al*. 2013). Suffering caused by care does not only involve patients but also relatives and caregivers involved in the actual situation and the healthcare organization (Eriksson & Svedlund 2007, Reader & Gillespie 2013). In addition, nurses in clinical practice need to be aware of factors that can affect the relation to the patients, including the patients’ trust in healthcare professionals and in the healthcare organization (Nilsson *et al*. 2015). Nurses have a key role in facilitating and mediating patient experiences and their difficult position in care situations (Jangland *et al*. 2011).

Another research perspective concerns how patients perceive that healthcare providers receive and react on complaints. Studies show that physicians who receive complaints about themselves may react strongly. Feelings of shock or panic, anger towards the patient, regret, depression and even suicide can follow a complaint (Jain & Ogden 1999, Cunningham 2004). This raises concerns about whether the physicians’ reactions impose a risk that the complaints lead to worse care rather than better (Cunningham 2004). It has also been described that patients can perceive healthcare organizations as closed systems that are governed by routines and that healthcare providers protect each other after complaints. Therefore, patients can fear that a complaint will make the situation even worse (Soderberg *et al*. 2012).

One more considerable field of research is based on the perspective of patient safety. Robinson *et al*. claim that patient satisfaction is an integral component for measures of healthcare quality (Robinson *et al*. 2014). If complaints are systematically reviewed, they provide indications of problematic trends in health care both on institutional and individual levels (Bismark *et al*. 2013, Reader *et al*. 2014). Although patient complaints are inherently personal, emotional and not intended to investigate failures systematically, they can provide unique knowledge that is hard to get in other measurements (Reader *et al*. 2014). In a mapping of the extent to which health care is influenced by complaints in Sweden, it was found that quality improvement measures were made in only 4·4% of cases (Hagelin 2007).

The aims of this study were to describe patient complaints to PAC and to examine possible associations between content in the statements from caregivers and reports of satisfaction/dissatisfaction from the complainants and finally to describe the complainants′ reasons for being dissatisfied after receiving a statement from the healthcare provider.

## The study

### Design

A retrospective and descriptive design was used to examine filed complaints.

### Method

This study was based on files drawn up at a Swedish County PAC during 2011. The procedures at the PAC are that patients or relatives contact the PAC by telephone, letter or e‐mail. To communicate a written complaint the complainants are asked to fill in and return a form. If the form is returned to the PAC, it is sent to the director of the clinic with a request for a statement. Then, the statement is returned to the complainant together with a brief questionnaire (Appendix 1). There is a wide spectrum in the content of the complaints. From minor comments on medical examination/treatment, unnecessary costs and long wait, to severe remarks on discrimination, abuse or big disappointment and outrage against the caregiver in the context of death of a close relative.

### Data collection

The material of the study consisted of 618 files at the PAC. The files varied in content but all contained complaints or grievances from a patient or a relative. There were both complete and incomplete files. *Complete files* consisted of a written complaint, a statement from the healthcare provider and a questionnaire response from a patient or a relative. In the questionnaire, the respondents were asked to answer whether they were satisfied with the healthcare providers’ statement or not. If they were dissatisfied, they were asked to state a reason. *Incomplete files* consisted only of notes from a phone call or a letter to the PAC and/or files where questionnaire responses were lacking. In 2011, there were 221 complete files. All these were included. In addition, 397 incomplete files (from 1 January 2011–31 August 2011) were included. The first step of the study was a pilot study of 200 cases. The first author (CS) and the third author (EMA) in cooperation read the complaints to determine variables for background factors. Next, the content of statements and questionnaire responses were analysed to establish content categories. This was made by CS and EMA through joint discussions and reflections until agreement was achieved. The material was then used to develop three reading guides/protocols: One for the details of the complaints (Appendix 2), one guide for significant/typical categories in statements from healthcare providers and one for the questionnaire responses both with 13 categories (Appendices 3 ‐ 4). In the second step of the study, data were collected according to the three reading guides/protocols. The files were read by CS and to calibrate assessments and when uncertainty aroused also by EMA and decisions about categorizations were made in cooperation.

### Analysis

In the first step of the study, a qualitative approach was used to categorize content of the texts in statements and questionnaires. Quantitative methods were then used in the second step. Data were analysed with descriptive statistics and differences between groups were tested with Chi‐squared test. Bivariate odd ratios and confidence intervals were calculated. We chose a value of *P* < 0·05 and 95% CI as statistically significant. Statistical analyses were performed using IBM Statistical Package for the Social Sciences (SPSS) version 22.0.

### Ethics

Filed cases at the PAC are covered by confidentiality. The results of the study are reported only on a group level and individuals cannot be identified. The regional ethical vetting board in Stockholm approved the study (Dnr: 2012/956‐31/5).

## Results

The study included 618 complaints at different clinics from either a patient (67%) or a relative (33%). Most complained by phone (71·5%) and the remaining by letter or e‐mail. Most complaints concerned patients aged 20‐79 (80%). Patients under the age of 20 or over the age of 80 were represented in roughly equal proportions. A greater proportion of complaints concerned female patients (57%) than male patients and more women than men lodged complaints regarding themselves (60% vs. 40%). The most frequent category of relatives who lodged complaints were mothers (30%) followed by daughters and wives. The share of complaints at different clinics agrees well with the share of patient visits at each hospital clinic while primary care had a smaller proportion. In some cases, there were lodged complaints about more than one clinic, for example, both primary care and a hospital clinic.

The reasons for complaints were categorized into three groups: (1) complaints concerning healthcare/medical treatment 365 (59·1%); (2) complaints concerning organization/rules 227 (36·7%); and (3) complaints concerning attitudes/communication 214 (34·6%). More than one category may have been used. Women represented a larger proportion of those who left complaints concerning healthcare/medical treatment (60%) and attitudes/communication (58%) while the proportion of women and men were equal in the category of organization/rules. In 26% of the cases, the complainants had received information about the patient insurance and in 11·5% of the cases about the possibility to complain to National Board of Health and Welfare.

The content in the statements from the healthcare providers was categorized. One statement could relate to several categories. Associations between categories and the complainants’ satisfaction as reported in the questionnaire (being dissatisfied or being satisfied) were assessed. In all, 221 complainants responded to the questionnaire. Of those, 72% were dissatisfied and 28% were satisfied. More women (64%) than men were satisfied with the healthcare providers’ statement. More relatives (61%) than patients who had lodged complaints about themselves were satisfied.

Four of thirteen categories were associated with being dissatisfied. These were: ‘Disagree/defend themselves’, ‘Only an explanation of normal routine’, ‘Clinics refer to/blame each other’ and ‘Brief statement’. When the statement included that the healthcare provider ‘Disagreed/defended themselves’, the association with being dissatisfied was statistically significant and the odds ratios almost three times higher for being dissatisfied (OR = 2·83, *P = *0·023). When the statement did not include any of these four categories a statistically significant association with being satisfied was found and the odds ratio was lower than 1, which means that this constitutes a factor against being dissatisfied (OR = 0·36, *P = *0·001) (Table 1).

**Table 1:** Associations between categories in statements from caregivers (more than one category may have been mentioned) and reports of dissatisfaction/satisfaction presented as percentages, OR with 95% CI and *P* values

	Proportion of satisfied (*n = *61)	Proportion of dissatisfied (*n = *160)	OR	95% CI	*P* value
Categories, most frequently reported by the dissatisfied					
Disagree/defend themselves	10%	24%	2·83	1·13−7·09	0·023*
Only a description of adherence to standard routines	23%	34%	1·70	0·86−3·35	0·14
Involved clinics refer to(blame) each other	2%	6%	3·97	0·50−31·72	0·30
Brief statement	7%	10%	1·57	0·50−4·91	0·60
Combinations of above four categories					
None of the above mentioned	64%	39%	0·36	0·20−0·67	0·001**
One of the above mentioned	31%	48%	2·08	1·11−3·88	0·02*
Two or more of the above mentioned	5%	12%	2·74	0·79−9·60	0·14
Categories, most frequently reported by the satisfied					
Admit to have made mistakes but claims that this didn't affect the outcome.	26%	15%	0·49	0·24−1·01	0·08
Refer to internal problems (organizational)	21%	14%	0·58	0·27−1·25	0·22
Will review the incident and explains how for example routines will be modified	16%	10%	0·56	0·24−1·32	0·24
Regret	69%	62%	0·72	0·39−1·35	0·35
Combinations of above four categories					
None of the above mentioned	20%	32%	1·90	0·93−3·86	0·10
One of the above mentioned	38%	42%	1·18	0·64−2·16	0·65
Two or more of the above mentioned	43%	27%	0·49	0·27−0·91	0·03*

*P < 0·05; **P < 0·01.

OR, odds ratio; CI, confidence intervals.

Another four categories were associated with being satisfied: ‘Acknowledging mistakes’, ‘Refers to internal problems’, ‘Shall review the incident and/or describe how a routine will be changed’ and ‘Regrets’. When statements combined two or more of these categories, the association with being satisfied was statistically significant with an odds ratio that indicate that these combinations of two or more categories constitutes a factor against being dissatisfied (OR = 0·49, *P = *0·03) (Table 1).

After receiving a statement from healthcare providers, the complainants responded to a questionnaire (Appendix 4). As previously mentioned, 72% (*n = *160) reported that they were dissatisfied with the statements they had received from the healthcare providers. The two most frequent causes for reports of dissatisfaction were that the healthcare provider ‘did not tell the truth’ and ‘insufficient information’ (26% respectively). As reasons for dissatisfaction, 17% reported: ‘Not being listened to/not being taken seriously’, 16% reported ‘medical questions unanswered’ and 14% reported ‘Insufficient care’.

## Discussion

The most challenging result in this study was that less than 30% of the complainants were satisfied with the healthcare providers’ statements about the complaints. On the basis of the present results, it can be assumed that Swedish PACs do not fully succeed in their two main assignments, which are to help and support patients and to contribute to quality improvement in health care.

The results show that the most common area for complaints concerned health care/treatment followed by complaints about attitude/communication and organization/rules. These results correspond with results from a recent systematic review of 59 international studies (*n = *88,000 patients) (Reader *et al*. 2014). To identify problems concerning patient safety, the authors proposed a coding taxonomy with subcategories at three different levels. They claimed that ‘rigorous analysis of patients′ complaints’ (Reader *et al*. 2014, p. 678) are essential for identifying problems in patient security.

This study identified four categories of statements that were associated with dissatisfaction. These categories (‘Disagree/defend themselves’; ‘Only an explanation of normal routine’; ‘Clinics refer to/blame each other’ and ‘Brief statement’) are related to failings that mainly concern institutional and professional standards, in other words, to ‘procedure neglect’ (Reader & Gillespie 2013). However, the respondents’ perceptions for not being satisfied (‘did not tell the truth’, ‘insufficient information’; ‘not being listened to/not been taken seriously’, ‘unanswered medical questions’ and ‘insufficient care’) also reflect that procedure neglect is closely related to ‘caring neglect’. Caring neglect refers to ‘failings in care that are below the threshold of being proceduralized//yet lead patients, family and the public to believe that staff are unconcerned about the emotional and physical wellbeing of patients’ (Reader & Gillespie 2013) (p. 8 of 15). This implies that instead of providing alleviation, support, security or participation care causes confusion, frustration and abandonment (Arman *et al*. 2004, Nordgren *et al*. 2007). Suffering from care means that patient's dignity and human value is violated by activities or actions that involve neglect or exercise of power (Eriksson *et al*. 2006). Thus, strength of the present result is that it highlights the relation between procedure neglect and caring neglect. This underscores the importance of how healthcare providers receive and respond to complaints.

Insufficient statements from healthcare providers can have several explanations. To receive a complaint is often distressing and the criticism can fill the person, the healthcare provider, with strong emotions (Robinson *et al*. 2014). Previously, it has been described that a majority of physicians who receive a patient complaint react negatively, which can make it more difficult to admit mistakes (Cunningham 2004). Another reason for insufficient statements may be that Swedish healthcare providers are not fully aware of the PACs’ role for handling patient complaints. Statements are often characterized by denial or defence, which can be interpreted as the healthcare providers perceiving the PAC as an authority that can punish in the form of warnings against the clinic or against individual staff members. Moreover, health care has been described as a culture where infallibility prevails (Ödegård & Wallgren 2007), which can be a reason for strong reactions. A culture that strives for infallibility can affect the degree of honestly reported adverse events or managers’ active involvement (Ödegård & Wallgren 2007). Thus, by striving for infallibility, there is a risk that failures are denied or that patients who turn to the PAC risk being dismissed and this can be assumed to reduce patient confidence in health care (Ödegård & Wallgren 2007). Consequently, healthcare organizations need leadership that ensures both that staff receive support and that complaints are received and handled in a constructive way that lead to quality improvement (Robinson *et al*. 2014, Piper & Tallman 2015). It is reasonable to assume that complainants have at least two goals with their complaints: to obtain redress and to prevent that other patients experience the same flaws in health care (Jangland *et al*. 2009, Piper & Tallman 2015). None of these objectives are met if healthcare providers respond only in terms of disagreement, defence, explaining normal routines or referring to other clinics. It can also be assumed that a complaint will lose its meaning if it does not result in, for example, a change in routines. In turn, this means that the PACs’ assignments to help the patient and to reinforce patient safety will not be fulfilled. Finally, one reason for insufficient statements can be a lack of organizational structures for handling complaints from patients. At an organizational level, there is a need for a systematic analysis of the causes of complaints and what institutions and/or members of staff they are directed to (Bismark *et al*. 2013, Reader & Gillespie 2013). The aim was to find appropriate support and training. At clinic/unit level, it is necessary to ensure a leadership providing an ethical culture for receiving complaints and criticism with patients′ best in focus (Piper & Tallman 2015).

It is also important to acknowledge that patients who decide to lodge a complaint are filled by strong emotions. They may feel disappointment, grief, humiliation, anger, bitterness and sometimes even hatred (Kent 2008), which can contribute to distrustfulness. A patient who lodges a complaint can perceive that the healthcare providers protect each other instead of objectively investigating the event (Kent 2008). Thus, the complainant can perceive that he or she is at risk of being refused care by healthcare providers who suppose that the patient mistrusts the healthcare provider. Nurses have unique opportunities to listen and respond to patients’ experiences of health care and suffering. However, they need awareness about how patients conceive care. This can be organized at their work‐place in, for example, supervised reflective ethical seminars or group sessions (Berglund *et al*. 2012). In addition, systematic training of communication skills is valuable (Allan *et al*. 2015). Also, to ensure good quality of care and patient satisfaction, evaluation of nurses’ tasks regarding information, clinical pathways or guidelines and other routines can be conducted (Nilsson *et al*. 2015).

The present result identified four categories associated with being satisfied. When two or more of these categories were combined, or if none of the four identified categories were used, the associations were statistically significant. This may be explained in different ways. First, it can be assumed that complainants are more satisfied if they receive detailed statements. Thus, it is not receiving a statement that matters most; instead, it is a matter of what the statement contains. It has previously been described that complainants need to receive a personal explanation or apology from the involved healthcare providers (Skar & Soderberg 2012, Soderberg *et al*. 2012).

Second, when the statements contained information about what actions it actually led to (i.e. the category ‘Shall review the incident and/or describe how a routine will be changed’), there were associations with being satisfied. It can also be assumed that the complainant perceives that the healthcare provider has taken the complaint seriously, when expressed regret is a part of the statement although receiving solely an expression of regret not is enough. Stating that the clinic admits problems in their organizations may also be one way of showing that one is taking the complaint seriously in contrast to defending themselves. Thus, relatively simple measures can show the complainant, that the healthcare provider has considered the complainants’ physical and/or emotional wellbeing and is interested in helping the complainant (Eriksson & Svedlund 2007). In that way, complaints will result in a meaningful change for them (Robinson *et al*. 2014). Also from a patient safety perspective, this is an important issue. By recognizing mistakes and internal problems, the healthcare provider demonstrates that activities are critically examined and that they are prepared to change routines.

### Limitations

The limitations of this study are the subjective nature of data and that the complaints filed at the PAC represent only a small share of the true number of dissatisfied patients and relatives. Furthermore, the data were collected from one single PAC, which might limit the ability to make generalizations. Another limitation is the questionnaire, since it only contained questions about whether the complainants were satisfied or not with the healthcare providers’ statements and the reasons for that. In future research, it would be interesting to ask also what has been helpful or not in the PAC's handling.

## Conclusions

The results of this study provide novel insights about patients’ complaints that can contribute to a scientific knowledge base and can be applied to facilitate quality improvements in clinical practice. The present results can be used in, for example, nursing education when teaching nursing ethics or for reflection and discussion among nurses in clinical practice. Procedure neglect, untrue explanations, lack of communication, blaming other clinics etcetera indicate that healthcare providers at times neither take their caring responsibility nor their obligation to learn from mistakes/incidents. Mistakes are inevitable, but healthcare providers need to learn a lesson from them, they need to listen and they need to respond in a helpful manner. To improve patient safety and contribute to quality improvement healthcare organizations should insure a leadership providing an ethical culture for receiving complaints and strive for transparency regarding complaints.

## Conflict of interest

The authors declare no conflicts of interests.

## Author contributions

Charlotta Skålen (CS) and Eva‐Maria Annerbäck (EMA) conceptualized/designed the study and collected/analysed data. CS, Lena Nordgren and EMA drafted the manuscript, and revised it for important intellectual content.

All authors have agreed on the final version and meet at least one of the following criteria [recommended by the ICMJE (http://www.icmje.org/recommendations/)]:

- substantial contributions to conception and design, acquisition of data or analysis and interpretation of data;
- drafting the article or revising it critically for important intellectual content.

## Appendix 1

### Questionnaire

TO YOU, WHO HAVE SUBMITTED COMPLAINTS TO THE PATIENT ADVISORY COMMITTEE:

In response to your communication to the patient advisory committee an investigation has been done. We have received responses from ….. … which are attached. We would be grateful if you answer the questions below and also leave comments with your answers. Please return the questionnaire in the enclosed envelope within 4 weeks, so that we can conclude your case.

**Table:**

1. Did you receive answers to your questions or comments?	
YES NO	
2. If no, what do you miss the answer?	
3. Do you feel satisfied with the answer?	
YES NO	
Write down any other comments here or use the back.	

## Appendix 3

### Nr: Reading guide for statements from healthcare providers

1. Brief statement
2. Regrets
3. Complain about treatment but receives a medical explanation
4. Will review the incident and describes for example how a routine will be changed
5. “Difficult patients”
6. Clinics refer to one another
7. Refers to internal problems (organizational)
8. Only an account of the normal routine and that it was followed
9. Encourages continued contact
10. Informs that the complaint constitutes a deviation
11. Other _______________________________________
12. Disagree with/defend themselves
13. Acknowledging mistakes but says that it has not affected the final result

## Appendix 4

### Nr: Reading guide for questionnaire responses

Not satisfied

1. Missing someone who takes responsibility for the incident
2. The absence of financial compensation
3. Unnecessary expenditures
4. Medical questions unanswered
5. Not listened to/not taken seriously
6. Disagree with about the disease/diagnosis
7. Waiting time/not adequately treated in a timely manner
8. Don′t tell the truth
9. Administrative misses/messy organization
10. Freedom of choice and health care guarantee
11. Lack of care
12. Insufficient information
13. Other _______________________________________
